# Temperature Changes and SEM Effects of Three Different Implants-Abutment Connection during Debridement with Er:YAG Laser: An Ex Vivo Study

**DOI:** 10.3390/ma12223748

**Published:** 2019-11-14

**Authors:** Jacek Matys, Umberto Romeo, Krzysztof Mroczka, Kinga Grzech-Leśniak, Marzena Dominiak

**Affiliations:** 1 Dental Surgery Department, Medical University, 50-425 Wroclaw, Poland; kgl@periocare.pl (K.G.-L.); marzena.dominiak@wp.pl (M.D.); 2 Private Dental Practice, Lipowa 18, 67-400 Wschowa, Poland; 3 Department of Oral Sciences and Maxillofacial Surgery, 00161 Rome, Italy; umberto.romeo@uniroma1.it; 4 Institute of Technology, Pedagogical University, 30-084 Krakow, Poland; krzysztof.mroczka@up.krakow.pl; 5 Department of Periodontics, School of Dentistry, Virginia Commonwealth University, Richmond, VA 23298, USA

**Keywords:** one-piece implant, peri-implantitis, peri-mucositis, titanium, two-piece implant

## Abstract

The study aimed to evaluate a temperature increase in, and damage to, titanium implants during flapless laser debridement. The study analyzed 15 implants with various implant–abutment connections: a two-piece implant (n = 4) with a screw abutment (IA—Implant–Abutment) and a one-piece implant with a ball type fixture (BTF, n = 4) or fix type fixture (FTF, n = 4). The implants were placed in porcine mandibles 2 mm over a bone crest to imitate a peri-implantitis. The implants were debrided in contact mode for 60 s with a Er:YAG laser at fluence of 9.95 J/cm^2^ (G1 group: 50 mJ/30 Hz); 19.89 J/cm^2^ (G2 group: 100 mJ/30 Hz); 39.79 J/cm^2^ (G3 group: 200 mJ/30 Hz), or a scaler with a ceramic tip (G4 control group: 4 W/20 Hz). The temperature was measured with thermocouples at implant and abutment levels. The damage in the titanium surface (n = 3, non-irradiated implants from each type) was assessed using SEM (Scanning Electron Microscopy). The temperature increase at the implant level for the laser was higher at IA in contrast with FTF and BTF. (p < 0.05) The temperature change at the abutment level was lower for the scaler in contrast to Er:YAG laser at FTF. (p < 0.0002) Er:YAG laser didn’t increase the temperature by 10 °C at 100 mJ/30 Hz and 50 mJ/30 Hz. Based on SEM analysis, cracks occurred on the surface of two-piece implants and were more pronounced. Cracks and the melting of the titanium surface of two-piece implants cleaned with Er:YAG laser at 100 or 200 mJ were observed. The specimens treated with the ultrasonic scaler with a plastic curette showed the remaining dark debris on the titanium surface. We recommend using Er:YAG laser at 50 mJ/30 Hz during flapless implants debridement.

## 1. Introduction 

Rehabilitation of patients needing dental implant restorations is now a predictable method of treatment; however, failures occur [1]. Bacterial infection of dental implants is the most common reason for peri-implant mucositis or peri-implantitis, and causes implant loss [2,3,4].

Peri-implantitis and peri-implant mucositis are connected with a bacterial biofilm occurrence, which is composed mainly of gram-positive facultative cocci and rods bacteria [5,6,7]. The inflammation process around an implant surface needs to be removed, because it leads to crestal bone loss and decreases in long-term implant survival rate. Decontamination of infected titanium implant surface and removal of bacterial biofilm can be achieved by surgical and nonsurgical means [8]. Surgical therapy involves resective or regenerative techniques for advanced peri-implantitis in shallow or deep intrabony defects, respectively [9,10]. Furthermore, in early and moderate stages of peri-implantitis, nonsurgical techniques, e.g., air-powder abrasion, citric-acid, chlorhexidine application, ultrasonic, manual debridement, implantoplasty or laser treatment, can be applied [11,12,13,14,15,16,17,18,19,20]. However, the complex architecture of the implant makes establishing a decontamination protocol difficult. Thus, traditional tools such as curettes or ultrasonic scalers used alone are inadequate to ensure proper treatment of an implant surface contaminated with bacterial biofilm [21]. Moreover, mechanical debridement of the titanium surface carries the potential risk of damage to the implant surface [22,23,24].

A useful tool for nonsurgical implant surface debridement and detoxification are erbium lasers [13]. Recent studies showed the benefits of Er:YAG laser use operating in a non-contact mode for soft tissues [25,26], for bone [27,28] and especially for bacterial biofilm eradication [29] without damage [30] to the dental implant titanium surface or overheating of peri-implant tissue. Additionally, a laser beam allows for the cleaning of even a small area of the implant threads which are inaccessible to mechanical instruments [31]. Therefore, our previously published study [18] showed differentiation in temperature increase for the two most common titanium grades used in implant dentistry when irradiating with a diode and Er:YAG lasers. We noted that implants composed of grade IV titanium heat up much faster than grade V titanium implants composed of titanium, aluminum, and vanadium alloy. Moreover, an analysis of implant temperature increase concerning implant diameter revealed significant differences between both laser types in terms of a correlation between a rise in temperature and a decrease in implant diameter. Thus, in this study, we compared the temperature gradient after laser debridement for various implant to abutment connection types at the same implant titanium grade.

It was proven that Er:YAG lasers could play a significant role in decontaminating an infected implant surface [32]. However, particular attention should be paid to prevent overheating of the bone and damage to implant surface when using these devices during surgery [33]. Due to direct bone-implant-contact and the unique composition of the soft tissue in the implant neck area, the blood flow in this area is reduced, which increases the risk of thermal injuries being transmitted by the implant to the bone tissue. Eriksson et al. [34,35] found in a series of studies that increasing the temperature of bone tissue by 10 °C for 60 s causes permanent changes in the bone structure. Therefore, a tissue temperature gradient (∆Ta) below 10 °C should be regarded as optimal and safe.

Our study aimed to evaluate the implant temperature increase, depending on the type of implant-to-abutment connection and various laser parameters, during implants’ debridement using Er:YAG laser and ultrasonic scaler in nonsurgical approaches. Furthermore, damage to the implant titanium surface was analyzed.

## 2. Materials and Methods

### 2.1. Samples Collection

**Figure 1:**
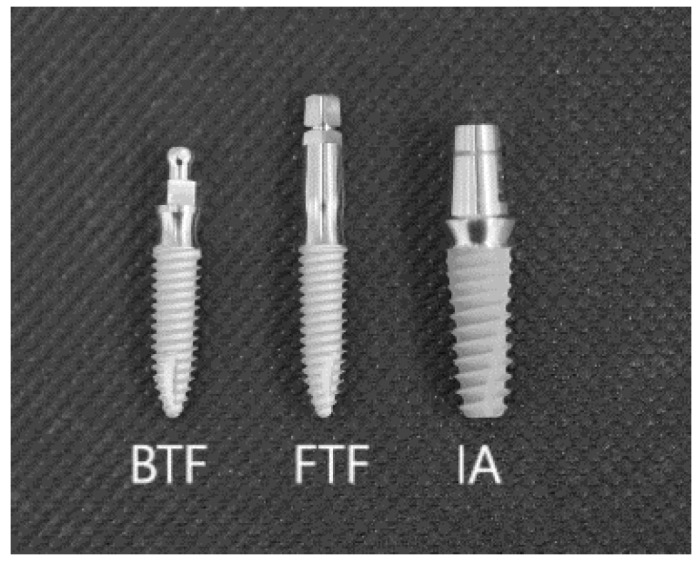
Type of grade 4 titanium alloys implants used in the study (BTF—ball type fixture; FTF—fix type fixture; IA—implant-to-abutment connection of two-piece implants).

Twelve heads of a 10-month-old male pigs, breed: Złotnicka Biała, intended for consumption, and which had been obtained from a butcher, were used in this study. We applied two different devices: an Er:YAG laser (LightTouch, Syneron, Yokneam, Israel) and an ultrasonic scaler with a plastic tip (PM200, EMS, Nyon, Switzerland) for the debridement of three various implant types (n = 15); a two-piece implant with a screw-type implant-to-abutment connection (IA, n = 5) (Slimline, Dentium Co., Seoul, Korea), and two types of one-piece implant with a ball type fixture (BTF, n = 5) and a fix type fixture (FTF, n = 5) (Slimline, Dentium Co., Seoul, Korea) (Figure 1).

### 2.2. Sample Preparation

Twelve mandibles (n = 12) were prepared from the pig heads, then washed under tap water and left for 4 h before the research was commenced. In every mandible, preparation of the soft tissues between the canine (C) and first premolar (P1) gave access to the mandibular alveolar ridge. Ethical approval was not required for this animal ex-vivo study.

### 2.3. Surgical Procedure

**Figure 2:**
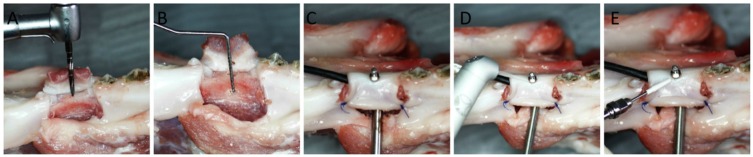
Surgical and measurement procedures used in the study. (**A**) The implant bed preparation. (**B**) Prepared implant bed and a hole with a diameter of 3 mm at mid-height of the buccal side of the implant bed for a Thermocouple Probe placement. (**C**) The implant, with two probes placed at mid-height and collar level of the implant. (**D**) Er:YAG laser with a sapphire tip before debridement procedure. (**E**) The scaler with a plastic tip before debridement procedure.

**Figure 3:**
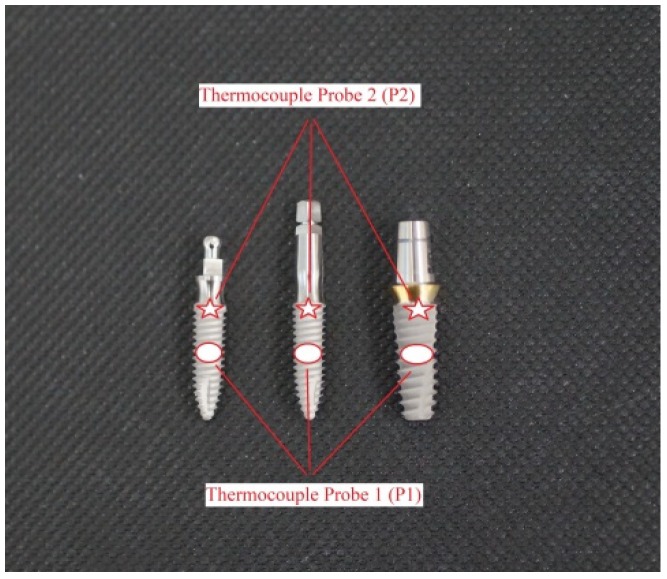
The position of the thermocouples P1 (red ellipse) and P2 (red arrow) attached to the implant surface.

In the study area of the mandible, a full-thickness flap had been made by two vertical and one horizontal cuts using a 15 C scalpel blade. The soft tissue flap was detached, and using drills, three various implant beds with a length of 12 mm were prepared, according to manufacturer protocol. A hole (3 mm in diameter) was drilled in each mandible at mid-height of the buccal side of the implant bed, with a trephine bur to place a K Thermocouple Probe 1 (P1), type TP-02 (Zhangzhou Weihua Electronic Co., Zhangzhou, China). In each implant bed, a corresponding implant composed of grade IV pure titanium was placed: a two-piece implant (diameter of 3.6 mm) with standard straight screw abutment (IA), a one-piece implant (diameter of 3.0 mm) with BTF or a one-piece implant (diameter of 3.0 mm) with FTF. The implants with abutments were placed in porcine mandibles of 2 mm over a bone crest to imitate a periimplantitis. The abutments were exposed through small cuts in the soft tissue, and the flap was then repositioned and sutured to the soft tissue using non-absorbable suture (Dafilon^®^, Braun, Germany). The second, a K Thermocouple Probe 2 (P2), type TP-02 (Zhangzhou Weihua Electronic Co.), was attached to the border of the implant and abutment. Debridement procedure was performed by placing the laser or scaler tip in a pocket, 2 mm below soft tissue margin, and moving the tip up and down from the medial to the distal implant/abutment area for 60 s (Figure 2 and Figure 3).

### 2.4. Study Groups

The study specimens (n = 12) were divided into four groups: G1 (n = 3), G2 (n = 3), G3 (n = 3), G4 (n = 3).

G1 Group: Er:YAG laser (LiteTouch^®^, Syneron Dental, Yokneam, Israel), operation mode for hard tissues (HT) was used, power: 50 mJ, frequency: 30 Hz, energy density per pulse: 9.95 J/cm^2^, water spray cooling (100%): 30 mL/min., size of the tip: 0.8 mm × 17 mm, distance: contact mode.

G2 Group: Er:YAG laser (LiteTouch^®^, Syneron Dental), operation mode for hard tissues (HT) was used, power: 100 mJ, frequency: 30 Hz, energy density per pulse: 19.89 J/cm^2^, water spray cooling (100%): 30 mL/min., size of the tip: 0.8 mm × 17 mm, distance: contact mode.

G3 Group: Er:YAG laser (LiteTouch^®^, Syneron Dental), operation mode for hard tissues (HT) was used, power: 200 mJ, frequency: 30 Hz, energy density per pulse: 39.79 J/cm^2^, water spray cooling (100%): 30 mL/min., size of the tip: 0.8 mm × 17 mm, distance: contact mode.

G4 group (control): ultrasonic scaler (PM200, EMS, Nyon, Switzerland with a plastic tip at 4 W/20 Hz.), power: 4 W, frequency: 20 Hz, water spray cooling: 30 mL/min (Table 1).

The rest of the implants (n = 3), which were not inserted into the pig’s bone, were used as a control in Scanning Electron Microscopy (SEM). 

### 2.5. Measurement Procedure

The specimens were placed in a container with water at a room temperature of 22 °C for 20 min; the temperature was monitored with a Medicare Clinical Products (MCP) Gold mercury thermometer (Medicare Products Inc., New Delhi, India). The temperature of the implant and abutment were measured employing a calibrated digital Thermometer CHY802W (CHY. Firemate Co., Tainan City, Taiwan) with the temperature probes of the K Thermocouple Probe, TP-02 type (Zhangzhou Weihua Electronic Co.). The measurement error was 0.3 °C. A thermo-conductor paste ART covered the thermocouples.AGT-057 (AG Thermoplasty, Sokoly, Poland) to ensure proper thermal flow. The thermal conductivity of the paste was 0.243 Cal/g K. The temperature rise after 60 s of the laser irradiation, and scaling were recorded. Every measurement was taken three times, and the obtained mean subjected to statistical analysis. The maximum temperature was noted a few seconds after implant debridement for 60 s with laser or scalar when the temperature had reached a steady state.

### 2.6. Scanning Electron Microscopy

A total of 15 implants were assessed in SEM analysis. The implants were sputter-coated with approximately 30 nm gold. Scanning electron microscopy (SEM, acceleration 10 kV, Spot Size 40 and 50 nm) evaluated the damage of the implant titanium surface. The analysis was conducted by a scanning electron microscope (JEOL6610LV, JEOL, Akishima, Japan) with a secondary emission detector (SEI, JEOL). 

### 2.7. Statistical Analysis

The obtained outcomes were subjected to statistical analysis utilizing Statistica 12 (StatSoft^®^, Tulsa, OK, USA) software. The mean increases in temperature of the implants and abutments have been assessed using the one-way ANOVA test. Pair comparisons were carried out based on the Tukey posthoc test at significance levels p = 0.05.

## 3. Results

### 3.1. Temperature Rise at Implant Level (P1 Thermocouple)

The analysis of temperature rise, measured by a P1 thermocouple at the implant level, revealed a significantly lower temperature gradient for the each abutment type after irradiation using Er:YAG laser at 50 mJ/30 Hz, in contrast to Er:YAG laser at 100 mJ/30 Hz and 200 mJ/30 Hz (p < 0.0002). The highest mean temperature increases of 6.54 ± 0.96 °C, 5.04 ± 0.96 °C, 4.35 ± 0.54 °C were found at 200 mJ/30 Hz for two-piece implant abutment (IA), fix type fixture (FTF) and ball type fixture (BTF), respectively (p < 0.05).

Furthermore, the temperature increases measured by P1 thermocouple after laser irradiation were higher at IA’s connection type when compared with FTF and BTF (p < 0.05).

However, for the scaler, we obtained significantly greater temperature increases at the BTF in comparison with FTF and IA. (p < 0.05) (Table 2)

### 3.2. Temperature Rise at Abutment/Implant Level (P2 Thermocouple)

The analysis of temperature increase, measured by a P2 thermocouple at the abutment level, revealed a significantly lower temperature gradient for the two-piece implant (IA) after irradiation using Er:YAG laser at 50 mJ/30 Hz, as compared with Er:YAG laser at 100 mJ/30 Hz and 200 mJ/30 Hz (p < 0.002). However, for the scaler at FTF (G4), the temperature increase was significantly lower in contrast to laser irradiation (G1, G2, G3) (p < 0.0002).

The highest mean temperature rises of 5.86 ± 0.46, 7.62 ± 0.74, 10.67 ± 1.14 were found at 200 mJ/30 Hz for two-piece implant (IA), FTF and BTF, respectively (p < 0.0002).

The temperature rises in the G3 and G4 groups, measured by P2 thermocouple, were higher at BTF when compared with FTF and IA connection types (p < 0.05). However, in the G1 and G2 groups the highest temperature increase measured using the P2 thermocouple was noted for the FTF in comparison with IA (G3) and BTF (G4) (p < 0.05) (Table 3).

**Figure 4:**
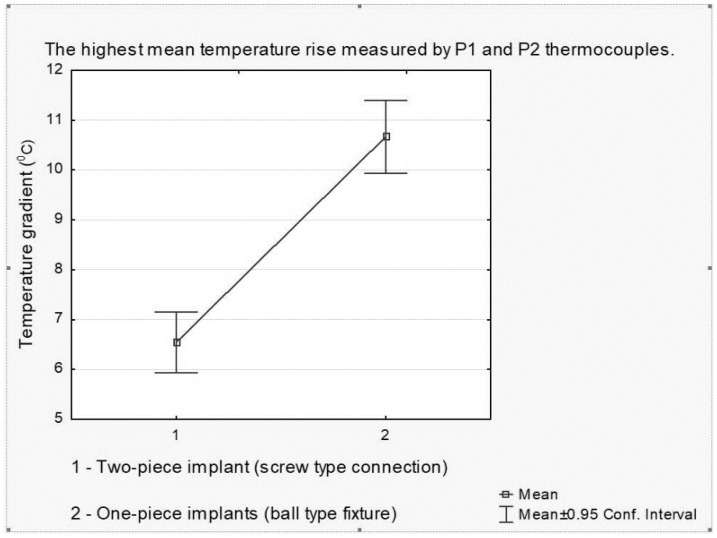
The highest mean temperature rise measured by P1 (at implant’s level) and P2 (at abutment’s level) thermocouples. The temperature rise by the critical 10 °C (10.67 + 1.14 °C) was noted for the ball type fixture (BTF) at the abutment level after lasing at 200 mJ/30 Hz and was significantly higher compared with FTF and IA.

The analysis showed that, after 60 s of flapless debridement with Er:YAG at 200 mJ/30 Hz, the temperature increase by the critical 10 °C (10.67 ± 1.14 °C) was noted for the ball type fixture (BTF) of the one-piece implant at the abutment level (P2 thermocouple). However, the highest mean temperature growth (6.54 ± 0.96 °C) measured by the P1 thermocouple at the mid-side of the two-piece implant was below 10 °C (Figure 4).

### 3.3. SEM Analysis

**Figure 5:**
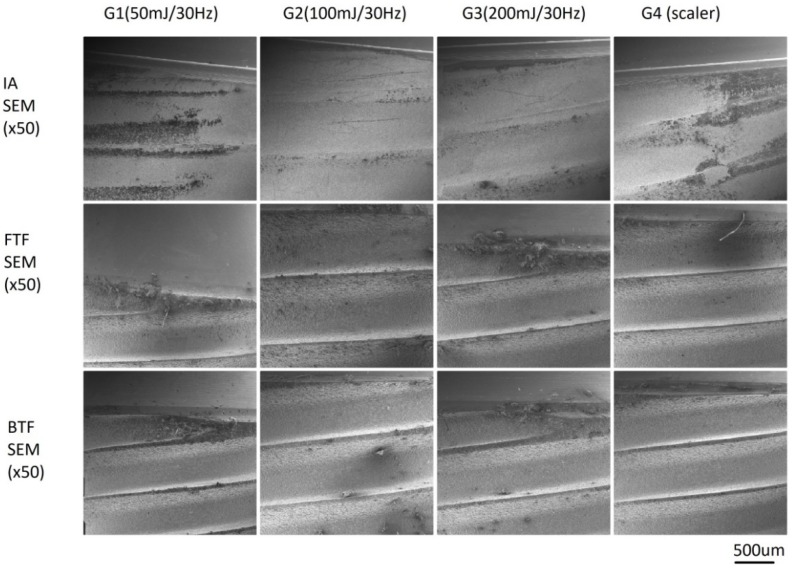
Damage to the SLA surface of implant treating by laser with different parameters (G1, G2, G3) and a scaler (G4) in contact mode. Scanning Electron Microscopy (SEM) ×50. The minor damage (scratches, cracks) on the surface of titanium implants was found after debridement with both Er:YAG laser and scaler for all groups. However, less was found for the scaler on the one-piece implants with ball type fixture.

**Figure 6:**
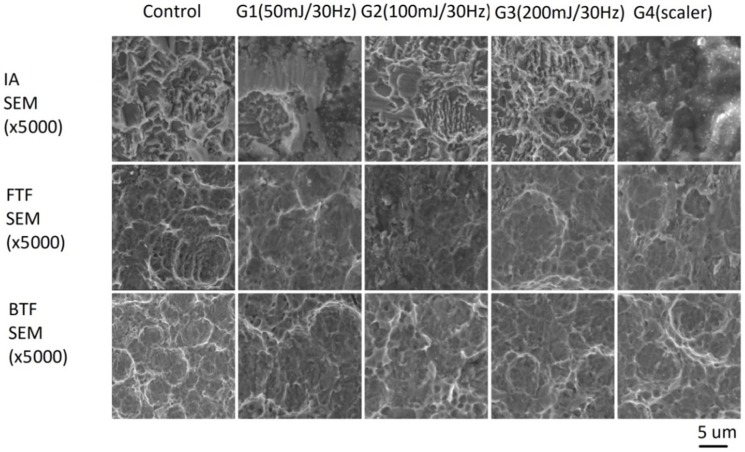
Damage to the SLA surface of implant treating by laser with different parameters (G1, G2, G3) and a scaler (G4) in contact mode. SEM ×5000. Noticeable damage (cracks, melting) to the titanium surface of two-piece implants cleaned with Er:YAG laser at 100 or 200 mJ was observed. Also, the specimens treated with the ultrasonic scaler with plastic curette showed the remaining dark debris on the titanium surface.

To test the damage to implants’ titanium surface, SEM analysis was conducted. The main finding was that all samples debrided with the Er:YAG laser and the scaler showed minor damage (scratches, cracks) on the titanium surface of various implants. However, less damage was found when debriding one-piece implants with the scaler. Two-piece implants seem to be more sensitive to scratches during contact flapless debridement for all methods/devices (Figure 5). Furthermore, noticeable damage (cracks, melting) to the titanium surface of two-piece implants cleaned with the Er:YAG laser at 100 or 200 mJ was observed. Also, the specimens treated with the ultrasonic scaler with a plastic curette showed the remaining dark debris on the titanium surface (Figure 6).

## 4. Discussion 

The application of lasers for dental implants’ debridement has been investigated with regard to different wavelengths and protocols [18,29,30,36]. The present study contributes to the existing knowledge by testing the use of an Er:YAG laser operating in contact mode and an ultrasonic scaler with a plastic tip in a flapless debridement of one-piece and two-piece four grade titanium implants. The main finding of the present study was that Er:YAG laser at indicated parameters (100 mJ/30Hz and 50 mJ/30Hz) supports debridement of two-piece and one-piece implants with temperature rise at collar and at a mid-high level of implants below the critical 10 °C. Furthermore, the SEM analysis of each sample debrided by the Er:YAG laser and scaler indicated minor damage (melting, cracks) to the implants titanium surface.

The prime aim of the present study was to evaluate the temperature gradient at mid-height of implants during their debridement using various devices and treatment protocols. In our study we obtained the highest mean temperature increases measured at implant levels of 6.54 ± 0.96 °C, 5.04 ± 0.96 °C, 4.35 ± 0.54 °C at 200 mJ/30 Hz/60 s for a two-piece implant with a standard abutment (IA), fix type fixture (FTF) or ball type fixture (BTF), respectively. The results of the present study were below the critical temperature growth by 10 °C, which causes irreversible damage to the peri-implant bone [34,35]. In 2002, Kreisler et al. [37,38] investigated temperature changes at the implant–bone interface during simulated implant surface decontamination with Er:YAG laser (pulse energy: 60–120 mJ, 10 Hz, 0.6–1.2 W). They concluded that the temperature has not increased by 10 °C after 120 s of irradiation, which also confirmed the safeness of the laser device. The efficiency of Er:YAG laser was also confirmed by Taniguchi et al. [30], who presented its ability to remove calcified deposits from contaminated titanium microstructures without causing substantial thermal damage at pulse energies below 30 mJ/pulse (10.6 J/cm^2^/pulse) and 30 Hz with water spray. In our study, we tried to establish the safe parameter for the Er:YAG laser operation without a significant increase in the implant temperature (above 10 °C), and found them to be 50 mJ (1.5 W), 100 mJ (3 W), 200 mJ (6 W).

Different conclusions to ours have been presented by Geminiani et al. [39] and Leja et al. [40]. They concluded that Er:YAG lasers induced temperature growth above the critical threshold of 10 °C only after 10 s of irradiation. It should be emphasized that the study conducted by Lejla et al. [40] compared the temperature rise of the implants placed in pig ribs after lasing with air or air/water cooling, respectively. Unfortunately, the authors have not described the cooling parameters in detail (mL/min). The authors also pointed out that the 250 mJ Er:YAG laser reached a calculated temperature of 31.4 °C with air cooling, but only 4.4 °C with air/water cooling [35]. Hence, we can readily perceive that the waterflow capacity has a crucial influence on the final temperature gradient of the irradiated implants. 

The second issue discussed in the study was the assessment of temperature increase at the implant’s collar during debridement of various abutments. In our present study, we found the temperature gradient over the critical threshold of 10 °C (10.67 + 1.14 °C) during the debridement procedure only for the one-piece implant with BTF at 200 mJ and 30 Hz. This result does not vary much from the critical threshold of 10 °C indicated by Eriksson et al. [34,35] in their two studies in the rabbit model. In turn, the study of Trisi et al. [41] in a human model showed that low-density bone seems to be frailer to heat-induced damage than high-density bone. Consequently, temperature rises in the cortical bone or collar part of the implant (measured by P2 thermocouple) slightly higher than the critical threshold should not cause irreversible thermal damage. However, our present research was an ex vivo study with characteristic limitations, e.g., various chemical structures and the biological features of the ex vivo specimens in contrast to in vivo tissue, mainly due to the lack of blood circulation. Thus, our findings should also be confirmed in the human in vivo model and using the implants of various manufactures.

Furthermore, the mean temperature gradient was higher for the fix type fixture as compared with implant–abutment interphase of two-piece implants. The one-piece implants differ from the two-piece systems in their constant implant–abutment connection [42]. This fact could influence the heat dissipation around various abutments. The distribution of heat during laser irradiation depends on lasing device parameters, diameter, and grades of titanium implants, but also is conjugated with peri-implant bone density [18,43,44,45]. Therefore, taking into account the susceptibility of the crestal peri-implant bone to resorption, the use of a mean power below 6 W (200 mJ/30 Hz) or an increase in the water flow over 20 mL/min is recommended to avoid the risk of thermal damage in this particular area.

Another goal of our study was to evaluate damage to the implant’s surface using SEM analysis. We found laser debridement caused minor damage to the titanium structure of one-piece implants with BTF and FTF, based on SEM analysis, while pronounced cracks and melting occurred in two-piece implants. In our present study, we cleaned the surface of the implants by placing the tip of the Er:YAG laser and ultrasonic scaler below the soft tissue margin remaining in contact with the treated implants. In this procedure, we debrided the implant surface without an operators’ sight control; this can lead to higher risk of damage to the titanium surface, due to the generation of high photomechanical effects between the titanium surface and a sapphire tip transporting energy to the target area. Moreover, in the literature, there are studies [20,42,43] assessing the degree of damage in the implant titanium surface under the effect of scalers with a polymer coated tip. The main conclusion of these studies was that the polymer scalers are efficient in cleaning the titanium implant without any damage to its surface. In our study, we have shown that the ultrasounds caused damage to two-piece implants microstructure and evoked the implants’ surface darkening. The possible explanation of the dark debris covering the surface of the implant is a remaining material which was split from a plastic tip after the treatment. Taniguchi and colleges [30] also confirmed this finding.

A particular focus in the present research was to assess the Er:YAG laser effects during debridement of the implant–abutment interface as a non-surgical therapy of peri-implantitis. Several previous reports have confirmed a high decontamination potential of lasers in vitro and in vivo studies [36,45,46,47,48,49,50]. However, the various laser parameters and protocols used in different studies did not enable the finding of clear and safe recommendations in the non-surgical treatment of peri-implantitis employing Er:YAG laser [51]. The high decontamination potential of Er:YAG laser in infected titanium implants was found at parameters of 100 mJ/pulse, 10 Hz ( =12.7 J/cm^2^) [45]. The efficiency of peri-implantitis treatment was also confirmed for Er:YAG laser at 100 mJ/pulse, 10 Hz with water cooling by a significant improvement of the clinical parameters, e.g., BOP = bleeding on probing, CAL = clinical attachment level, PD = probing depth in 6-month follow-up [36]. Furthermore, Sennhenn-Kirchner et al. [47] reported a nearly complete removal of fungal cells with Er:YAG laser using the same laser parameters (100 mJ/pulse 10 Hz).

Both decontamination potential and irrigation efficiency are important during peri-implantitis therapy. Many authors confirmed the ability of Er:YAG laser to remove bacterial biofilm and decontamination of titanium surface at 60–100 mJ/10 Hz [30,38,45]. However, the removal of inflamed tissues from peri-implant pocket using Er:YAG laser by debridement of the implant and abutment surfaces is crucial in determining a significant clinical benefit. To enhance the water irrigation pressure in a peri-implant pocket, the ratio of pulse energy and frequency is critical. In a different study, we recommended inducing water irrigation following the photoacoustic phenomena by the use of the energy/frequency ratio of 50 mJ/50 Hz [52]. The erbium laser used in this study allowed the induction of visible water agitation by photoacoustic effect at 100 mJ/30 Hz, without the temperature rise by 10 °C for all abutment types. This energy also is sufficient for bacterial biofilm removal. Hence, in our opinion, the use of the Er:YAG laser at 100 mJ/30 Hz in debridement of peri-implant pocket could be safe and efficient.

All these facts make variable decontamination protocols necessary. Therefore, it is even more important to know the effects of laser irradiation on the implant surface, and the temperature increases of different implant types and materials which are safe for clinical use. The various possible settings of Er:YAG laser, enables effective treatment of peri-implantitis, but further studies are needed to better understand its thermal impact on both the intraosseous components and prosthetic superstructures.

## 5. Conclusions

The result of this study can be summarized as follows:

- The Er:YAG laser debridement of two-piece and one-piece implants did not exceed the implant temperature by 10 °C at 100 mJ/30 Hz and 50 mJ/30 Hz;
- One-piece implants heat up faster than two-piece implants during Er:YAG laser irradiation at the implant’s collar area;
- Based on SEM analysis, the cracks and melting that occurred on the surface of two-piece implants were more pronounced compared to one-piece implants with the ball type fixture (BTF) and fix type fixture (FTF);
- Debridement of one-piece implants with a ball type fixture using Er:YAG laser at 200 mJ/30 Hz or more should be avoided. However, our findings should also be confirmed in the human in vivo model and using the implants of various manufactures.

We recommend using the Er:YAG laser with an energy/frequency ratio of 50 mJ/30 Hz during non-surgical therapy of peri-implantitis.

## Figures and Tables

**Table 1:** The parameters of devices used in the study.

Study Groups	Handpiece	Distance (mm)	Energy (mJ)	Frequency (Hz)	Power (W)	Spot (mm)	Fluence (J/cm^2^)	Time (s)	Power Density (W/cm^2^)	Cooling (mL)
G1	Laser-in-*Handpiece*	contact	50	30	1.5	0.8	9.95	60	298.5	30
G2	Laser-in-*Handpiece*	contact	100	30	3	0.8	19.89	60	593.7	30
G3	Laser-in-*Handpiece*	contact	200	30	6	0.8	39.79	60	1193,7	30
G4	Scaler	contact	-	20	4	0.9	-	60	-	30

**Table 2:** The mean temperature gradient at implant level (P1 thermocouple). The temperature rose significantly with an increased laser fluence. (p < 0.0003) The temperature rise measured at the implant level after laser irradiation was higher at IA’s connection type, in contrast to FTF and BTF. (p < 0.05).

Study Groups	Thermocouple P1∆Ta (°C) IA (I) (Mean ± SD)	Thermocouple P1∆Ta (°C) FTF (I) (Mean ± SD)	Thermocouple P1∆Ta (°C) BTF (I) (Mean ± SD)	P Value
Group 1	1.55 ± 0.55	1.07 ± 0.27 ^+^	0.86 ± 0.46 *^+^	IA vs. FTF, BTF p < 0.05 FTF vs. BTF p > 0.05
Group 2	3.62 ± 0.74 *	4.42 ± 0.38 *	2.37 ± 1.37 ^+^	IA, FTF vs. BTF p < 0.01 IA vs. FTF p > 0.05
Group 3	6.54 ± 0.96 *	5.04 ± 0.96 *^+^	4.35 ± 0.54 *^+^	IA vs. FTF, BTF p < 0.0004 FTF vs. BTF p > 0.05
Group 4	1.15 ± 0.54	1.57 ± 0.27 ^+^	2.43 ± 0.23 ^+^	IA vs. FTF vs. BTF p < 0.05
P value	G1 vs. G2, G3 p < 0.0002 G4 vs. G2, G3 p < 0.0002 G1 vs.G4 p > 0.05	G1 vs. G2, G3 p < 0.0002 G4 vs. G2, G3 p < 0.05 G1 vs. G4 p > 0.05	G1 vs. G2, G3, G4 p < 0.0003 G2 vs. G3 p < 0.0002 G3 vs. G4 p < 0.0002 G2 vs. G4 p > 0.05	

* Indicate significant differences between laser and control group G4 (ultrasonic scaler); ^+^ Indicate significant differences between the two-piece implant (IA) and one-piece implants (FTF, BTF).

**Table 3:** The mean temperature gradient at abutment level (P2 thermocouple). The temperature rose significantly with an increased laser fluence. (p < 0.0003). The temperature rise by the critical 10 °C (10.67 + 1.14 °C) was noted only for the ball type fixture (BTF) at 200 mJ/30 Hz. The temperature increase was significantly lower for the scaler (G4) in contrast to Er:YAG laser at FTF. (p < 0.0002).

Study Groups	Thermocouple P2∆Ta (°C) IA (A) (Mean ± SD)	Thermocouple P2∆Ta (°C) FTF (A) (Mean ± SD)	Thermocouple P2∆Ta(°C) BTF (A) (Mean ± SD)	P Value
Group 1	0.95 ± 0.55 *	3.47 ± 0.27 *^+^	2.40 ± 0.35 ^+^	IA vs. FTF vs. BTF p < 0.05
Group 2	2.55 ± 0.58	5.50 ± 0.89 *^+^	3.12 ± 0.74 ^+^	FTF vs. IA, BTF p < 0.0002 FTF vs. BTF p < 0.0002
Group 3	5.86 ± 0.46 *	7.62 ± 0.74 *^+^	10.67 ± 1.14 *^+^	IA vs. FTF vs. BTF p < 0.05
Group 4	2.00 ± 0.89	2.13 ± 0.23	2.97 ± 0.27 ^+^	BTF vs. IA, FTF p < 0.003 FTF vs. BTF p < 0.003 IA vs. FTF p > 0.05
P value	G1 vs. G2, G3, G4 p < 0.002 G2 vs. G3 p < 0.0002 G3 vs. G4 p < 0.0002 G2 vs. G4 p > 0.05	G1 vs. G2 vs. G3 vs. G4 p < 0.0002	G3 vs. G2, G4 p < 0.0002 G1 vs. G3 p < 0.0002 G1 vs. G2, G4 p > 0.05 G2 vs. G4 p > 0.05	

* Indicates significant differences between laser and control group G4 (ultrasonic scaler); ^+^ Indicates significant differences between the two-piece implant (IA) and one-piece implants (FTF, BTF).
